# The Treatment of Acute Inflammation by Passive Congestion. Bier's Method

**Published:** 1907-12-28

**Authors:** Charles W. Cathcart

**Affiliations:** Surgeon, Royal Infirmary, Edinburgh.


### THE TREATMENT OF ACUTE INFLAMMATION BY PASSIVE CONGESTION.

### Bier's Method.

By CHARLES W. CATHCART, F.R.C.S., Surgeon, Royal Infirmary, Edinburgh.

The plan of tying an elastic bandage on a limb above an acutely inflamed part is, at first sight, so unlike any method of treatment which has been found successful in the past, that no one need wonder that many surgeons rejected the method at first without giving it serious thought. The anti-phlogistic treatment of centuries has left its mark on our views of to-day. We cannot easily change our attitude, and, with Professor Bier, look upon inflammation not as a purely mischievous process, but as an agent which should be directed and made use of. For this reason if the passive congestion treatment were still merely at the stage of a suggestion very few would have the courage to try it. The method, however, has already been largely and successfully employed. For many years Professor Bier tested his method quietly, and only a few years ago drew much attention to it. His paper read at the meeting of the German Surgical Congress in 1904, was accompanied by illustrative cases and made a deep impression. Ever since that time the method has been finding a wider and wider circle of enthusiastic supporters. The second edition of "Hyperæmie als Heilmittel" was published in 1905; in the present year, 1907, the much enlarged fifth edition has appeared, and both the demand for the book and the need for its enlargement have been due to the increasing interest in the sections dealing with acute inflammation.

Just as "a good wine needs no bush," so a good treatment is its own best recommendation. Those who wish to try the method should begin by treating only the less severe cases of inflammation by passive congestion, while maintaining for the more severe cases the methods of treatment with which they are familiar. As the surgeon gains confidence in the new treatment he will extend his use of it. Among the cases considered suitable to begin with may be included (1) all quite recent septic inflammations, especially when they are limited in extent; (2) sub-acute and less severe inflammatory affections, whether recent or not, and (2) gonorrhœal, pyæmic, and acutely suppurating joints before the tendon sheaths and bones have become involved. Perhaps the best plan would be for the surgeon to test the treatment upon an inflamed scratch, or similar slight inflammation on his own person.

An elastic bandage for a finger is easily made as follows: Select a rubber band an inch or two long, and to each end knot a piece of worsted thread or a narrow strip of bandage. Wind the elastic round the finger with only a slight stretching of the elastic, and fasten it by means of the free ends of the worsted. The constriction should hinder venous return and increase the swelling, but should not cause pain. It is often necessary to re-adjust the bandage several times before the requisite tension is reached. The congestion may be kept up for twelve or fourteen hours, either by day or night, as may be most convenient. In a case which responds to the treatment, the redness and tenderness rapidly subside and suppuration, which might have been expected under ordinary treatment, does not take place.

### General Considerations.

If this preliminary trial be found satisfactory, a more severe case may be dealt with. Suppose it be one of acute whitlow, extending along a tendon sheath to the wrist, and producing redness and swelling of the hand. A piece of Martin's rubber bandage should be bound round the upper arm. If the foot were affected the bandage should be applied to the thigh—*i.e.* always a considerable distance above the seat of mischief. The bandage is put on sufficiently tight to hinder definitely the venous return, while it only slightly diminishes the arterial flow. The pressure in acute cases is very light. Any accessible pulse below the bandage should be easily felt, and the limb should be warm. One great test of the proper degree of tightness is the relief of pain. Sometimes a good deal of patience is required before the exact degree of tightness which diminishes pain is attained. When this has been found the rapid relief of pain is very striking, and generally astonishes the patient's friends as much as it gratifies the patient. In a few cases, however, and these chiefly of gonorrhœal origin, pain may be increased at first, although it afterwards diminishes. Another test of the proper application of the bandage is the objective effect upon the limb. After a few minutes the whole limb begins to swell, and the "fiery red" colour, formerly limited, say, to the hand and wrist, will now extend right up to the elastic bandage, while the skin will also feel warmer. When the œdema is well established, serum escapes freely from any wound which may exist, and an appropriate amount of loose absorbent dressing will be required to soak it up. There must, however, be nothing tight round the limb below the bandage. Severe cases should be treated in bed. In bad cases the passive congestion is kept up for twenty-two out of the twenty-four hours, and this may be diminished by degrees to from eight to ten hours as the symptoms become less severe. The following points, however, have also to be attended to:—Great care and watchfulness must be exercised, especially in the first day or two. If the bandage be too slack, it does no good; if too tight, it will do harm. The bandage should be changed in position after it has been about ten hours in one place. Where there is much œdema the bandage may have to be tightened as the œdema is dispersed.

In parts of the body, such as the shoulder, where there is no room to vary the position of the bandage, Bier recommends that congestion should not be kept up for longer than eight to ten hours at a stretch, and that the part compressed by the bandage should be well washed with spirit at each interval. In the intervals of relaxation the limb should be raised so as to favour absorption of the effused lymph.

The progress of the case can be gauged by the patient's general condition and by the local appearances and symptoms. As a general rule the patient is not only soon eased of pain, but feels brighter and better. The pulse improves and the temperature drops. This reduction in the temperature occurs very quickly when the cause of the raised temperature is merely the absorption of poison from the inflamed area. A rise of temperature may then be observed during the intervals in which the bandage is relaxed. This, of course, is due to the setting free again of poison retained along with the serum. In proportion, however, as the treatment is successful in combating the suppurative inflammation, such rises of temperature diminish, since there is less poison generated in the local micro-organism factory. On the other hand, when the cause of elevated temperature is septicæmia or pyæmia, passive congestion can only influence the temperature indirectly by improving the condition of the focus from which the circulating blood has been contaminated, and such influence may, for a time at least, be inappreciable. At the affected place, the more acute the inflammation, the more easy it is to cause œdema and diffuse redness by the elastic compression. As the inflamed area improves by the use of the bandage, it responds less and less to constriction on the limb, until the reaction is almost as little as it would be in health. The surgeon, however, does not require to keep up the passive congestion as long as this. After convalescence has fairly set in, progress seems to go on faster without artificial aid. The same may, of course, be said of many other remedies besides that now under consideration. Any pus which may form should be let out, but the incision or incisions need not be so extensive as would have been necessary without passive congestion. Free incisions are only required when the circulation of the part is seriously impaired. Should blisters appear, either the bandage is too tight or suppuration has occurred which must be relieved.

### Some Illustrative Cases.

A good example of the value of the method is the following: Robert R., æt 40, a cowman from Bathgate, was admitted to Ward 18, Royal Infirmary, Edinburgh, on November 8, 1905, on account of pain and swelling in the right hand and forearm. About a fortnight before admission he had felt some pain in the thumb. This he thought was caused by the prick of a thistle, but he paid very little attention to the fact until November 5, when the pain became much worse, and kept him awake. An incision had been made into a swelling on the ball of the thumb and various dressings applied, but without giving relief. Hot poultices had eased him more than anything else, and by their means he had slept two hours on the night before he came in. When admitted, he was haggard and anxious looking; the hand was dark red in colour, and much swollen, and the redness and swelling extended irregularly up the forearm to the elbow. He was in great pain, and felt miserable. At 2 P.M. an elastic bandage was applied to the middle of the upper arm, and at 7 P.M. the following note was made: "The patient feels 'a guid bit easier since it has been pitten on.' The pain running up the inner side of the arm into the arm-pit has completely vanished. There is no throbbing in the hand nor stabbing as before. The fore-finger can be moved a little at the metacarpal joint. (He volunteered this information.) The improvement took place about three hours after the bandage had been applied, and since then he has had a couple of hours' sleep and seems refreshed, and his expression is not so anxious. There is great œdema of the hand and forearm below the bandage." On November 9 the patient said he "felt like a new man." From this time there was no further anxiety as to the spread of the mischief. A slough about the size of a hazel-nut separated from the pulp of the thumb on November 17, and an abscess was opened over the palmar aspect of the thumb on November 24. On December 12 the patient was discharged with the wounds nearly healed, although there was still some stiffness of the wrist and fingers.

### Another Case.

One of the most striking examples of the relief of pain by the use of passive congestion which I have met with in my own practice is the following: The case was one of so-called "gonorrhœal rheumatism." Mrs. X, aged 38, was suddenly seized with very severe pain in the left knee about the beginning of October. The pain came on without apparent cause while she was sitting in a friend's house. She had to go home in a car, and was carried upstairs on reaching her own house. There was very little rise of temperature or pulse, but the pain was extreme from the first until I saw her four weeks later. There was conclusive evidence that the patient had lately suffered from gonorrhœa, though she had paid no heed to the vaginal discharge, thinking it was only leucorrhœal. When I first saw the patient she was exhausted from pain and want of sleep, and had a careworn, anxious look. She could scarcely bear one to touch the knee, and was in great distress if the bed were shaken, or if one walked with a heavy step across the floor. The part was swollen, but the joint was not distended with fluid, and its temperature was not raised. A piece of elastic bandage was applied above the knee with sufficient firmness to produce slight venous obstruction. I had fortunately succeeded in getting the proper degree of passive congestion at the first trial, and in fifteen minutes there was a distinct feeling of relief. From that time she steadily improved, and made a good recovery, although a good deal of stiffness remained. I feared to awaken the disease again by forcible movement, so trusted to active and passive movements, massage, and hot fomentations. Her own medical attendant kindly sent me the following account of Mrs. X's case, which he allows me to publish: "For nearly a month I tried everything I knew to relieve the pain; extension, elevation, fomentations plain and medicated, liniments, iodine, etc. Internally, salicylates, potassium bromide, and potassium iodide, with no alleviation of the pain at all. Night after night I was sent for, and in the long run had to give morphia, and even this required large doses. I have never met with such an obstinate case, and she was being worn out from want of sleep and constant pain. Within a few hours—actually in the first hour—after putting on the elastic bandage, she felt eased, and the pain soon left her altogether, and has not returned. It acted like a charm, and gave relief at once, as well to the patient as to me."

Professor Bier narrates the details of a large number of cases of acute suppurative inflammation treated by this method, including septic wounds of joints, whitlows, osteo-myelitis, and such like. Other surgeons have obtained similarly good results in the majority of cases, although neither Professor Bier nor his followers claim that the method is infallible in checking sepsis. If carefully used by one who has learned the method by going from the less to the more severe cases, no harm will be done by employing it at the outset in every case.

### Conclusions.

For the last two years I have applied Bier's method to every case of acute inflammation of the extremities that I have met with in hospital or private practice, and have found it fail in only a small proportion of cases. Speaking generally, the kind of case in which the usual restraining action of the treatment is not seen is that in which the patient's powers of re-action against micro-organisms are unusually low. In cellulitis of the hand or forearm, accompanied by severe constitutional disturbance, but where suppuration is delayed, I have found that passive congestion gives little or no relief to pain, and does not seem to check the progress of the disease. Such cases are generally due to a streptococcus and leucocytosis is absent or slight. Other more typically suppurative cases, also due to a streptococcus, have, however, responded at once to the passive congestion. I have seen the same delay in suppuration and the same failure of passive congestion to check the disease in cases where the inflammation was due to a staphylococcus. The occasional failure of the treatment is probably due to a deficiency in the patient's resisting power relative to the virulence of the micro-organism. Where either the resistance is less or the virulence greater than usual the assistance to natural methods of cure given by the congestion will be insufficient. Possibly also the absence of relief of pain in such cases may be due to some difference in the quality of the blood serum. In a favourable case the relief of pain is so rapid that some mechanical effect is probably at work, surrounding the nerves with œdema or diluting the irritating poisons. If the serum tends to coagulate, for instance, these effects might be hindered or neutralised. So long as the bandage is carefully applied and the case watched, no harm will result, and it seems to me right that every septic affection of the extremities should have the benefit of the method.

The suction method of producing passive congestion which is applicable to the surface of the body has a more limited field, but is equally efficient.

